# Early Rising and Insanity

**Published:** 1896-03

**Authors:** 


### Early Rising and Insanity.

The great poets of the world have always sung in praise of sleep:

"—— sore labor's bath,
Balm of hurt minds."

They have always consistently opposed the habit of early rising, except in so far as they have depicted (writing by the light of the lamp) the glories of the morning and the suggestive promises of a beautiful sunrise. The scriptures are also on the side of prolonging slumber:

"Yet a little sleep, a little slumber, a little folding of the hands to sleep," says Solomon.

But the habits of modern civilization and the traditions of family life have been steadily set against these poetical and scriptural ideals. In whatever else men differ, or in whatever other way they err, it is agreed that the habit of early rising is seemly, advantageous and necessary.

The statement, therefore, by a prominent alienist and superintendent of a large insane asylum, that early rising promotes insanity comes with a terrible shock upon the serenity of established beliefs and long-cherished customs. Dr. Selden H. Talcott, of Middletown, N. Y., however, supplements this statement of his by arguments of no mean weight. He calls attention to the relative frequency with which farmers, their wives, daughters and sons become insane. The cause of this has heretofore been thought to be their isolated lives, their hard work, and, perhaps, the excessive use of pie and potatoes; yet these have never seemed quite adequate explanations, when one considers the fact that the farmer has constantly fresh air, abundant exercise, no undue excitement or mental strain, and relatively little alcoholism and syphilis. So that Dr. Talcott's view is, at least, a helpful one in the way of explaining the psychopathic tendencies of the ordinary agriculturist. He states that the excessive early hours of rising, which are customary among the farmers, and which hours are imposed upon their sons and daughters and wives, prevent a sufficient amount of rest. Growing children, he thinks, in particular suffer from "the artificial cut-off" which is applied so rigidly to their lives.

The suggestion of Dr. Talcott is valuable from another point of view, because the particular cause of insanity to which he refers is one that can be so easily and comfortably combated. It is not like the enforced abstinence from alcohol, and tea, and tobacco, high thinking, and all the other ordinary comforts or luxuries of civilization. The diminution of insanity by staying in bed later mornings is a task to which all will address themselves with pleasure, and we believe that every farmer's boy will have a sense of particular kindness toward the physician who has discovered this new method for preventing possible mental disorders. We might readjust the well-known lines of Sir William Jones, for the benefit of the farmer, somewhat as follows:

"Eight hours to sleep, to soothing pleasures seven,
Nine to the farm allot, and all to heaven."

—*Medical Record.*

